# 

**Published:** 1902-11-29

**Authors:** 


					the hospital. "The standard of highest purity."?Lancet. Nov. 29th, 1902.
CADBURY'8 ^ u CADBURY'8
COCOA pi \ COCOA
ABSOLUTELY FURS. ^ ? f* D*UO? O* AI.r4I.IH Om,
No. 844. Yol. XXXIII. [^Newspaper*.] SATURDAY,
Nov. 29th, 1902.
[SuhsoripticnTwma,] pRICB TWOPENCE.

				

